# Natural Human Infections With *Plasmodium cynomolgi* and Other Malaria Species in an Elimination Setting in Sabah, Malaysia

**DOI:** 10.1093/infdis/jiz397

**Published:** 2019-08-16

**Authors:** Lynn Grignard, Sonal Shah, Tock H Chua, Timothy William, Chris J Drakeley, Kimberly M Fornace

**Affiliations:** 1 Faculty of Infectious and Tropical Diseases, London School of Hygiene and Tropical Medicine, London, United Kingdom; 2 Centre for Climate Change and Planetary Health, London School of Hygiene and Tropical Medicine, London, United Kingdom; 3 Faculty of Medicine and Health Sciences, Universiti Malaysia Sabah; 4 Infectious Diseases Society Sabah-Menzies School of Health Research Clinical Research Unit; 5 Gleneagles Hospital, Kota Kinabalu, Malaysia

**Keywords:** malaria, *Plasmodium cynomolgi*, *Plasmodium knowlesi*, zoonosis, elimination

## Abstract

To determine the presence and species composition of malaria infections, we screened a subset of samples collected during a cross-sectional survey in Northern Sabah, Malaysia using highly sensitive molecular techniques. Results identified 54 asymptomatic submicroscopic malaria infections, including a large cluster of *Plasmodium falciparum* and 3 *P. knowlesi* infections. We additionally identified 2 monoinfections with the zoonotic malaria *Plasmodium cynomolgi*, both in individuals reporting no history of forest activities or contact with macaques. Results highlight the need for improved surveillance strategies to detect these infections and determine public health impacts.

Following the initial description of a large cluster of human infections with the zoonotic malaria *Plasmodium knowlesi* in 2004, *P. knowlesi* is now the main cause of human malaria in Malaysia [1, 2]. The state of Sabah on Malaysian Borneo has one of the highest burdens of *P. knowlesi* globally and reported 2030 clinical *P. knowlesi* cases in 2017, comprising 98% of all malaria cases reported [3]. Simultaneously, reports of nonzoonotic malaria cases have decreased dramatically, with only 23 cases of *P. falciparum* and 8 cases of *P. vivax* reported in Sabah in 2017. Malaysia aims to eliminate malaria by 2020, making it critical to identify remaining infections in the population.

The increased incidence of *P. knowlesi* has been associated with deforestation and environmental change, likely bringing people in closer contact with long-tailed and pig-tailed macaque reservoirs (*Macaca fascicularis* and *M. nemestrina*) [4]. These macaque species also carry other *Plasmodium* species, including the zoonotic species *P. cynomolgi* [5]. While human infection with *P. cynomolgi* had been demonstrated in laboratory studies, the first natural human infection with *P. cynomolgi* was identified in West Malaysia in 2011 [6]. A more recent study identified mixed *P. cynomolgi* and *P. knowlesi* infections in 5 clinical cases in Sarawak in Malaysian Borneo [7]. Subsequent studies identified multiple asymptomatic human infections with *P. cynomolgi* in Cambodia in 2015; however, no asymptomatic human infections have been described in Malaysia [8].

Within Sabah, high prevalences of *P. cynomolgi* and coinfections with *P. knowlesi* and *P. cynomolgi* have been described in both macaque and mosquito populations. In Northern Sabah, entomological investigations of the main vector of *P. knowlesi*, Anopheles *balabacensis*, identified relatively high numbers of mosquitoes biting humans were infected with *P. cynomolgi* [9, 10]. Similarly, *P. cynomolgi* is the most common and widely distributed malaria infection detected in macaque populations in Southeast Asia [5]. However, although molecular diagnostic methods are now used for all clinical malaria cases in Sabah, no human infections with *P. cynomolgi* have been described [3].

This study aimed to detect whether low-density asymptomatic malaria infections were present at a community level by using a subset of samples collected during an environmentally stratified cross-sectional survey in Northern Sabah [11]. Initial analysis estimated recent exposure to *P. knowlesi*, as measured by serology, as 5.1% (4.8%–5.4%); however, of 10 100 people sampled, only 9 *Plasmodium-*positive individuals were identified by polymerase chain reaction (PCR) and no individuals were microscopy positive. Species included *P. knowlesi*, *P. vivax,* and *P. malariae*, with no *P. falciparum* detected despite on-going transmission in the area. Although previous studies had identified geographical clustering of asymptomatic infections in individuals residing in villages where clinical cases were reported [12], all individuals initially identified by this study resided in different villages and households. To investigate whether additional infections were present in households and villages of infected individuals and to determine the species composition of these infections, we screened selected individuals with highly sensitive molecular techniques and sequencing.

## METHODS

A population-based cross-sectional survey was conducted in 4 districts in Northern Sabah in 2015, as described by Fornace et al [11]. Briefly, 10 100 individuals were sampled from 2849 households in 180 villages, in areas encompassing a wide range of ecologies. Blood samples were collected using finger prick blood sampling, with whole blood collected into precoated EDTA tubes (Becton-Dickinson, Franklin Lakes). Participants were interviewed on individual and household characteristics, including history of fever and treatment-seeking behavior. Based on the initial infections identified, we selected 876 samples for additional screening, including these infected individuals and survey participants residing in the same households or villages as these individuals and individuals residing in neighboring areas in addition to the 9 identified infections.

Whole-blood samples of selected individuals were extracted on a QIAsymphony SP/AS instrument (Qiagen, UK) using a QIAsymphony DNA Midi Kit (Qiagen, UK) and eluted in 200 µL of elution buffer. Extracted DNA was amplified with a genus-specific seminested PCR assay targeting 18S rRNA *Plasmodia*, using primers PlasmoM_N1F (5′-ATGGCCGTTTTTAGTTCGTG-3′) and PlasmoM_N1R (5′-TTGTGTTAGACACACATCGTTCC-3′) for nest 1 and PlasmoM_N2F (5′-GTTAATTCCGATAACGAACGAGA-3′) and PlasmoM_N1R for nest 2 [8]. Thermal cycling conditions were 25 cycles for nest 1 and 30 cycles for nest 2 at 94°C for 1 minute, 53°C for 1 minute, and 72°C for 1 minute. The amplified 18S rRNA gene was subsequently sequenced on the ABI 3730xl DNA analyzer, using the BigDye terminator v3.1 Cycle Sequencing Kit (Applied BioSystems) and primers from nest 2 of the 18s rRNA PCR.

## RESULTS

Individuals screened included 423 men and 453 women, with ages ranging from 5 months to 90 years (mean age 27). This included participants from 417 households in 87 villages across 4 districts. Of these, 54 individuals (6.2%; 95% confidence interval [CI], 4.7%–8.0%) were identified as *Plasmodia* positive by PCR. These included 2 *P. cynomolgi* infections, 3 *P. knowlesi* infections, 3 *P. malariae* infections, 1 *P. vivax* infections, 41 *P. falciparum* infections, and 4 infections that species could not be identified despite repeated attempts. No infected individuals reported a history of fever. Of the original 9 infections detected, sequencing results were consistent for *P. knowlesi* and *P. malariae* monoinfections and the mixed *P. malariae*, *P. vivax,* and *P. knowlesi* infection was identified as *P. malariae* and *P. vivax*. Both *P. cynomolgi* infections were detected by the initial PCR analysis; however, both infections were previously misdiagnosed, 1 as *P. vivax* and 1 as a mixed *P. knowlesi* and *P. vivax* infection.

**Figure 1.**
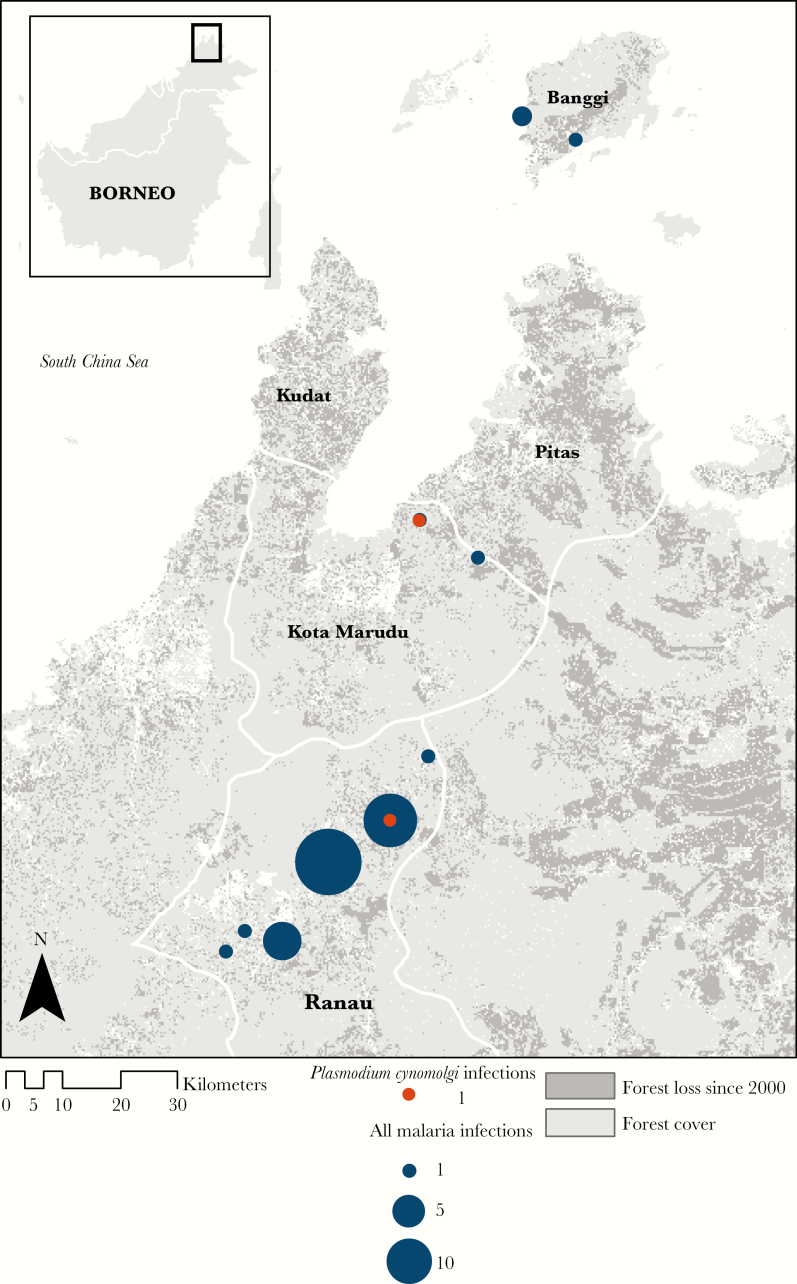
Study site and numbers of individuals identified with *Plasmodium cynomolgi* and other malaria infections per village.

Both *P. cynomolgi* infections were identified in adult men, aged 43 and 63. The individuals resided in different villages, 1 in Kota Marudu district and 1 in Ranau district (Figure 1). Both individuals described farming as their main occupation, primarily cultivating fruit and vegetables in close proximity to their households. Neither individual reported any forest activities or travel outside of the village in the past 30 days. Additionally, neither individual described any sightings nor contact with macaques. All *P. knowlesi* infections were also detected in adult men (ages 33, 46, and 47). Two individuals resided in the same village in Kudat district, with the other individual residing in Ranau. All individuals reported contact with long-tailed macaques around the house and village.

A large number of asymptomatic *P. falciparum* infections were identified despite no *P. falciparum* being identified by the previous study. Infections included 16 women and 35 men with a mean age of 31 years (range, 1–82 years). All *P. falciparum-*infected participants resided in Ranau district, including 12 household clusters with 2 to 4 *P. falciparum* positive individuals. Infections were also clustered at a village level, with 21 infections identified in 1 village out of 48 samples screened (43.8%; 95% CI, 29.8%–58.7%). An additional village in Ranau had 12 *P. falciparum* infections, 1 *P. cynomolgi* infection, 1 *P. vivax* infection, and 1 unspeciated *Plasmodium* infection and a third village in Ranau had 6 *P. falciparum* infections, 1 *P. knowlesi* infection, and 2 further *Plasmodium* positive infections that species could not be identified.

## DISCUSSION

This is the first description of natural human infections with *P. cynomolgi* in the Malaysian state of Sabah and the first description of asymptomatic *P. cynomolgi* monoinfections in Malaysia. Within this rapidly changing environment with close contact reported between human and macaque populations, there is a need to understand the burden and health implications of this zoonotic parasite and monitor changes in incidence, which could indicate an emergence similar to *P. knowlesi*. Although results from this study cannot be used to estimate prevalence or spatial distribution of malaria infections at a population level, the identification of areas with relatively high numbers of infections suggests on-going transmission at the time of the survey of both zoonotic and nonzoonotic malarias within an elimination setting.

Results highlight both the utility of molecular diagnostics and the challenges in applying these techniques. The initial survey screened all samples with a widely used seminested PCR assay targeting the *Plasmodium* genus developed for the 4 main nonzoonotic malaria species [11]. Although pooling may have reduced the sensitivity of these results, applying an assay specifically developed to target both zoonotic and nonzoonotic species detected substantially more infections. The failure to initially detect any *P. falciparum* infections may be due to the low sensitivity of the initial methods used or very low parasite densities. However, identifying the species composition of these infections remains challenging without sequencing confirmation. In contrast to other human *P. cynomolgi* infections identified, these cases were all monoinfections, although this may be due to the limited sensitivity of these methods to differentiate multiple species. A previous study in Malaysian Borneo did not detected *P. cynomolgi* infections within communities, despite specifically screening for this species [13]. As all infections were submicroscopic and asymptomatic, this suggests very low parasite densities and the need for improved diagnostics to detect these infections. Initial screening detected *Plasmodium* in both *P. cynomolgi-*positive individuals; however, both were misidentified by a routine PCR assay not targeting this species. This need is further emphasized by the relatively large number of *P. falciparum* infections detected in Ranau, an elimination setting with only 110 *P. falciparum* cases reported statewide in Sabah during the year of the survey.

Further studies are needed to assess the prevalence of asymptomatic infections and assess implications for current malaria elimination programs. While the public health impacts of *P. cynomolgi* remain unknown, this study illustrates the presence of naturally acquired human infections in individuals with no history of contacts with macaques or forest activities. Following the emergence of *P. knowlesi* within this region, innovative surveillance strategies are needed to detect zoonotic malaria infections and identify individuals and environments at risk.

## Notes

***Acknowledgments.*** We thank the Monkeybar project staff, partners at the Universiti Malaysia Sabah and Sabah Department of Health at the Malaysian Ministry of Health, and the study participants.

***Financial support.*** This work was supported by the United Kingdom Medical Research Council, Natural Environmental Research Council, Natural Environment Research Council, Economic and Social Research Council, and Biotechnology and Biosciences Research Council through the Environmental and Social Ecology of Human Infectious Diseases Initiative (grant number G1100796).

***Potential conflicts of interest.*** All authors: No reported conflicts of interest. All authors have submitted the ICMJE Form for Disclosure of Potential Conflicts of Interest. Conflicts that the editors consider relevant to the content of the manuscript have been disclosed.
